# Nutrition Status of People Experiencing Homelessness Residing in Temporary Accommodation in London

**DOI:** 10.1111/jhn.70024

**Published:** 2025-02-09

**Authors:** Hannah Style, Victoria Vickerstaff, Adrian Brown

**Affiliations:** ^1^ FEAST With Us (FEAST), Epsom London UK; ^2^ The Centre for Methodology and Evaluation Queen Mary University of London London UK; ^3^ Centre for Obesity Research University College London (UCL) London UK; ^4^ Bariatric Centre for Weight Management and Metabolic Surgery University College London Hospital NHS Trust (UCLH) London UK; ^5^ UCLH NIHR Biomedical Research Unit London UK

**Keywords:** dietary health inequalities, food insecurity, homelessness, malnutrition, nutrition status

## Abstract

**Objectives:**

London has the highest proportion of people experiencing homelessness (PEH) living in temporary accommodation in the United Kingdom. PEH have poorer health outcomes, greater dietary inequalities, and die younger than the general population. Despite this, little is known about the nutrition status of PEH. This study aimed to examine the dietary health inequalities experienced by PEH in London, specifically assessing malnutrition among PEH living in temporary accommodation.

**Methods:**

This was a prospective cross‐sectional study in 18 hostels in London. Participants were recruited from the temporary accommodation in which they resided through a combination of purposive, snowballing and convenience sampling. Demographic information was gathered, including age, gender, ethnicity and hostel of residence. The primary outcome was malnutrition risk assessed by the Malnutrition Universal Screening Tool (MUST), other outcomes included body composition, dietary intake and quality, mental health and food insecurity. Ethical approval was obtained from the University College London Ethics Committee (16191/006).

**Findings:**

Two hundred participants were recruited between July and December 2023. The majority were male (84.5%), were of White ethnicity (61%), with a mean (SD) age of 45.7 years (11.6) and a BMI of 23.4 kg/m^2^ (4.7). The median MUST score was 2 (interquartile range [IQR]: 0.0, 3.0), and 60% had a risk of malnutrition. The median mental health score was 6 (3.0, 10.0), with 55% having moderate to severe depression/anxiety. Median food security score was low (4.5 [(0.0, 8.0]), with 44% experiencing very low food security. The median dietary quality score was low (8.0 [6.0, 9.0]) with low intakes of energy, fibre, and micronutrients, including vitamin D, iron, folate, and calcium, with a higher intake of free sugars compared with UK dietary recommendations and intakes.

**Conclusion:**

This is the first study to show that PEH living in temporary residences had a high risk of malnutrition and experienced dietary inequalities related to poor diet quality and severe food insecurity. There is an urgent need for improved food environments, dietary quality of donated foods and improved nutrition screening and nutrition support provision for PEH in temporary accommodation. Findings could help inform policymakers, health services and food aid charities to set nutrition standards for temporary accommodation to promote the dietary health of PEH.

## Summary

- Malnutrition is prevalent among people experiencing homelessness alongside poorer diet quality, mental health, and high levels of food insecurity.
- Current nutritional screening practices appear insensitive to population‐specific nutrition risks and should be improved to promote prompt referrals for nutrition support.
- There is an urgent need for the development of nutrition guidelines and standards for people experiencing homelessness living in temporary accommodation.

## Graphical abstract

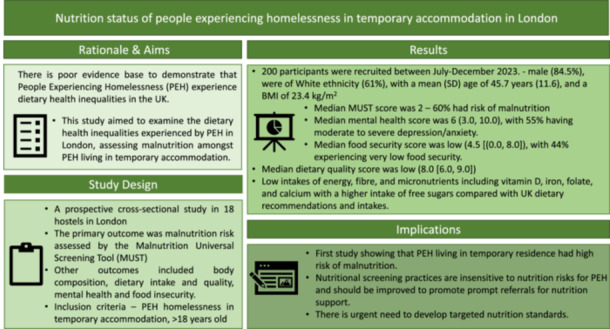

## 1 Introduction

Homelessness is broadly defined as the lack of reasonable conditions to live and is specified as ‘statutory’, ‘rough sleeping’, ‘hidden’ and ‘at risk’ [1]. Social Services and the Housing Executive can refer people experiencing homelessness (PEH) to temporary accommodation, including hostels, night shelters and council flats [2] where people do not have a fixed abode and rely on the government to provide shelter [3]. The number of PEH is increasing in the United Kingdom; notably, London has the highest proportion of PEH living in temporary accommodation (17.0 households per 1000) compared with the rest of England (2.3 households per 1000) [4], which has been suggested to be linked to the lack of affordable housing, poverty, unemployment and inadequate support services [5].

Data show that PEH experiences poorer health outcomes than the general population, often presenting with tri‐morbidity, the complex combination of physical and mental illness compounded by substance misuse [6]. Significantly, PEH experience premature death at 45–49 years [7], often from treatable causes, including gastrointestinal, respiratory and HIV‐related disease, and acute and chronic drug and alcohol dependence [8].

Homelessness has multiple causes, including poverty, systemic discrimination and inequality in access to services, and disproportionately low incomes in light of increasing cost of living prices [9]. Growing evidence suggests that compared to the general population, PEH are at higher risk of chronic food insecurity [10], defined as the lack of regular access to sufficient, safe and nutritious food. Food insecurity in PEH is associated with poorer mental health and reduced social ties [10], as well as dependence on unpredictable donated foods [11], which are often of poor dietary quality, exceeding dietary recommendations for fat, sugar and salt [12].

Those with lower incomes experience poorer nutritional status [13]. Evidence in England suggests that PEH experience poor dietary quality, including low intake of micronutrients, fibre, oily fish, fruit and vegetable intakes, and increased energy intake from saturated fat and nonmilk extrinsic sugars [14]. Furthermore, PEH may also be at higher risk of a compromised nutritional status [10], namely malnutrition, the functional and adverse effects related to an imbalance in energy intake and nutrient intake resulting in people experiencing both underweight and obesity [15]. However, understanding of the nutrient composition of PEH's diets is limited, with methodological limitations, population transience and heterogeneity, and poor access to health services making assessment challenging [16]. As such, there is a lack of evidence regarding the extent of malnutrition among PEH. With malnutrition risk being linked to poorer health outcomes [17], there is an urgent need to understand the degree of malnutrition in this vulnerable group.

This study aimed to address gaps in the literature by examining the potential dietary health inequalities experienced by PEH in London, specifically the prevalence and extent of malnutrition amongst PEH living in temporary accommodation. The study aimed to assess factors implicated in malnutrition risk, including food security, mental health, dietary quality and dietary nutrient composition of PEH in temporary accommodation. In doing so, the study aimed to highlight the malnutrition and dietary health inequalities experienced by this vulnerable population group and to assess what support is required to address these needs.

## 2 Methods

This was a prospective cross‐sectional study completed in collaboration between FEAST with the United States, a London‐based food insecurity charity, and University College London (UCL). Ethical approval was obtained from the UCL Ethics Committee (16191/006).

Between July and December 2023, participants were recruited from the temporary accommodation in which they resided through a combination of purposive, snowballing and convenience sampling. For clarity, temporary accommodation and hostels will be interchangeably used, as researchers exclusively visited hostels. Hostels for homeless adults in London boroughs, specifically Camden, Haringey, Islington, Enfield, Southwark, Barnet and Westminster, were contacted through existing FEAST with US networks, with a total of 18 consenting to participate. These hostels' funding was provided by either charities, local government authorities or a combination. Following the agreement, hostel staff were provided with an online briefing on the research protocol and asked for their collaboration to promote study participation in advance of study visits. Hostel managers were asked to provide feedback on the structure of the study visit and questionnaires to ensure they were appropriate for their residents. Advertising leaflets and posters were shared in hostel receptions, dining rooms where available, and communal areas with high footfall, with contact details of the research team.

Inclusion criteria were that in temporary accommodation, had sufficient English fluency to understand written and verbal instructions with appropriate adjustments, and capacity to provide informed consent after reading the participant information sheet at least 24‐h prior. Participants had the opportunity to ask questions of the research team and express interest during working hours. Before attending the study visit, hostel staff assessed participants' needs and potential intoxification. Participants were invited to attend a single study visit at their residing hostel. On the day of the visit, participants were briefed about the study and any questions were answered before taking written informed consent and commencing the study assessment.

Initially, demographic information was gathered, including age, gender, ethnicity, hostel of residence, self‐reported past medical history and current medication. Following this anthropometric data were collected. Weight was measured to the closest 0.01 kg and height to the closest 0.01 m using a mobile stadiometer (Seca 213). In addition, body composition was measured, including segmental muscle, body fat, bone and basal metabolic rate using bioelectrical impendence analysis (BIA) segmental scales (Tanita BC‐545N). Maximal muscle strength was taken from three hand grip strength (HGS) dynamometer readings (Dynamometer Takei 5001).

Subsequently, a series of online standardised questionnaires were completed to assess malnutrition risk, dietary intake and quality, mental health and food insecurity of the sample population. These were facilitated and completed by a member of the research team for the participants within an online survey to aid completeness.

### 2.1 Malnutrition Universal Screening Tool (MUST)

To assess malnutrition risk, the MUST was completed [18]. The MUST tool followed three steps: (1) calculating body mass index (BMI) by dividing weight by height in metres squared, (2) calculating self‐reported weight change as a percentage, and (3) marking acute disease score or period of 5 days with poor oral intake. Adding the score from these three steps gave a total malnutrition risk score. Malnutrition risk was defined as a score of 2 or more.

### 2.2 Dietary Intake and Diet Quality

Dietary intake and quality were assessed using two separate methods. The first method was the Short Form Food Frequency Questionnaire (SFFFQ) used to ascertain the Dietary Quality Score (DQS). The SFFFQ asked 20 questions to assess the consumption frequency of foods and food groups ranging from less than once per fortnight to multiple times per day. Additionally, the tool included lifestyle questions regarding physical activity and smoking habits. Data were entered into a downloaded spreadsheet with formulae devised by the tool authors, which gave a score between 1 and 3 for portions per week of fruit, vegetables, nonmilk extrinsic sugars (NMEs), fat and oily fish to calculate the total DQS. A score of three corresponds to meeting the UK dietary recommendations. A minimum of five indicated low diet quality, and a maximum of 15 indicated high dietary quality [19].

The second method was a single online multiple‐pass 24‐h recall, which was used to analyse the nutritional composition of participants' diet within the last 24‐h (Intake 24) [20]. A singular dietary recall was taken—instead of a repeated measure—following advice from the hostel managers and related to challenges of assessing dietary intake in PEH with population transience [16].

### 2.3 Patient Health Questionnaire 4 (PHQ4)

To assess participant mental health, the four‐item PHQ4 was used to measure the frequency over the past 2 weeks of symptoms of depression and anxiety taken from the DSM‐IV criteria. Questions used a four‐point Likert scale from ‘not at all’ to ‘nearly every day’ and were added to give a total score. Scores were categorised as none (0–2), mild (3–5), moderate (6–8), and severe (9–12) mental illness [21].

### 2.4 Food Insecurity

To assess the prevalence of food insecurity, the USDA Adult Household Food Security Survey [22] was used. Participants were asked 10 questions relating to food security in the past 30 days. The survey has a three‐item response scale: (1) ‘don't know’, (2) 0 = ‘only 1 or 2 days’ or (3) 1 = ‘almost every day’. Answers were added to give a total score; these were then categorised as high (0), marginal (1–2), low (3–5), and very low food security (6–10).

Participants were offered reasonable adjustments to increase participation, including utilising online translation software (Google Translate) for non‐English speakers, inviting support staff to attend the study visit at the discretion of the participant, and offering unfixed appointments to allow for extra time. Study visits took approximately 40 min, and participants were thanked and reimbursed with a £10 food voucher. With participant consent, the researcher provided a summary of clinical remarks to the hostel staff, with recommendations for onward referral if indicated. Data were collected online using RedCap Survey software and stored on a secured database on Data Safe Haven at UCL.

### 2.5 Anecdotal Discussions With Participants

During study visits, participants voluntarily raised dietetic challenges and issues in response to the questionnaires. Participants expressed highly emotive reactions including sorrow, despair and anger, especially regarding food insecurity. Researchers asked for permission to notify hostel staff and did not formally record or analyse observations as this would have been outside the remit of the study. However, these aided the context of the quantitative data and the discussion.

### 2.6 Data Analysis

Data were reviewed and cleaned to ensure data quality, and demographic data were summarised for parametric data using mean (standard [SD] and median [interquartile, IQR]) for skewed data. Categorical data were summarised using counts (percentages). Multivariable regression analysis was used to assess predictors of diet quality (DQS), risk of malnutrition (MUST), food insecurity and HGS. A stepwise regression model was run to identify the optimal multivariable model. All predictor variables were entered into the original model, and a backward elimination approach was implemented with variables being kept in if *p* < 0.1. Due to the limited numbers of ethnic minority groups within the study, to assess the impact of ethnicity on outcomes a dichotomous was created into white ethnicity (1) and ethnic monitory groups (0). Coefficients, 95% confidence intervals (CI) and *p*‐values were reported for outcomes. The assumptions of each model were checked and met. Statistical analysis was performed in Stata (version 18). Statistical significance was defined as a *p*‐value < 0.05.

## 3 Results

### 3.1 Demographic Characteristics

Between July to December 2023, 209 temporary accommodation residents were approached from 18 hostels across North, North‐West, Central and South London, and a total of 200 completed the study visit (95%). Reasons for exclusion include being unable to participate related to embarrassment about unclean feet and not wishing to stand on scales barefoot (*n* = 4), scales not reading high weight (n = 2), intoxification (*n* = 2), and mental health relapse at the time of the study visit (*n* = 1). The mean age was 45.7 years (SD 11.6), and the majority were male (*n* = 169; 84.5%) and self‐reported White ethnicity (*n* = 122; 61%; Table 1).

**Table 1:** Participant characteristics.

Characteristics	*n* = 200
Gender, *n* (%)	
Male	169 (84.5)
Female	31 (15.5)
Age, years (SD)	45.7 (11.6)
Ethnicity, *n* (%)	
Asian/Asian British	13 (6.5)
Black/Black British	33 (16.5)
Middle Eastern/Middle Eastern British	4 (2.0)
Mixed White/Black/Black British/Other	28 (13.5)
White British/Irish/Other	122 (61.0)
Anthropometry	
Weight, kg (SD)	70.9 (16.2)
BMI, kg/m^2^ (SD)	23.8 (4.7)
Body composition	
Muscle mass, kg (SD)	52.5 (10.1)
Muscle mass percentage, % (SD)	75.0 (8.7)
Fat mass, kg (SD)	15.6 (9.1)
Fat percentage, % (SD)	21.0 (9.2)
Visceral fat score, median score (IQR)	7.0 (4.0, 10.5)
Bone mass, kg (SD)	2.8 (0.5)
Bone mass percentage, % (SD)	4.0 (0.47)
BMR, kcal per day (SD)	1615.0 (307.8)
BMI categories, *n* (%)	
Underweight	20 (10)
Healthy weight	101 (50.5)
Overweight	52 (26.0)
Obesity class I	21 (10.5)
Obesity class II	6 (3.0)
Malnutrition universal screening tool score, median (IQR)	2.0 (0.0, 3.0)
Score 0, *n* (%)	70 (35.5)
Score 1, *n* (%)	10 (5.0)
Score 2–6, *n* (%)	120 (60.0)
Step 1 score (BMI), median (IQR)	0 (0.0, 0.0)
Step 2 score (weight loss), median (IQR)	0.0 (0.0, 1.0)
Step 3 score (disease), median (IQR)	2 (0.0, 2.0)
Handgrip strength, kg (SD)	35.8 (10.0)
Dietary quality score, median (IQR)	8.0 (6.0, 9.0)
Patient Health Questionnaire 4, median (IQR)	6.0 (3.0, 10.0)
None, *n* (%)	50 (25.0)
Mild, *n* (%)	40 (20.0)
Moderate, *n* (%)	41 (20.5)
Severe, *n* (%)	69 (34.5)
Food security, median (IQR)	4.5 (0.0, 8.0)
Very low, *n* (%)	88 (44.0)
Low, *n* (%)	35 (17.5)
Marginal, *n* (%)	24 (12.0)
High, *n* (%)	53 (26.5)

Abbreviations: %, percentage; BMI, body mass index; IQR, interquartile range; kg, kilograms; kg/m^2^, kilograms per metre squared; *n*, number; SD, standard deviation.

### 3.2 Malnutrition

The median MUST score was 2 (IQR: 0.0, 3.0), and a high proportion of participants presented with a score of 2–6 (*n* = 120, 60%), indicating a high prevalence of malnutrition risk. The median scores for steps 1 (BMI) and 2 (weight loss) of the MUST were both 0 (IQR: 0.0, 0.0 and 0.0, 1.0 respectively), whilst the median score for step 3 was 2 (IQR: 0.0, 2.0), indicating that disease and/or periods of limited oral intake were prominent factors in the prevalence of malnutrition in PEH in temporary accommodation.

### 3.3 Anthropometrics, Body Composition, and Functional Strength

Participants' mean weight was 70.9 kg (SD 16.2) with a mean BMI of 23.4 kg/m^2^ (SD 4.7). Half of the participants were living with a healthy BMI (*n* = 101, 50.5%), over a quarter lived with overweight (*n* = 52, 26.0%), and a smaller proportion lived with underweight (*n* = 20, 10%), and obesity (*n* = 27, 13.5%). Participants' mean basal metabolic rate was 1615.0 kcal/day (SD 307.8).

Participants' mean fat mass was 15.6 kg (SD 9.1), equating to 21.0% body weight (SD 9.1), of which the median visceral fat was 7.0 kg (IQR: 4.0, 10.5). Mean muscle mass was 52.5 kg (SD 10.1), representing 75.0% (SD 8.7) body weight and participant mean bone mass and percentage bone mass were 2.8 kg (SD 0.50) and 4.0% (SD 0.47), respectively. The mean maximal HGS of the three consecutive measures was 35.8 kg (SD 10.0). Low HGS readings indicate sex‐specific sarcopenia among 19% (*n* = 38) of the participants [23], of which 7% (*n* = 14) were not identified by MUST as being at risk of malnutrition.

### 3.4 Mental Health

The median PHQ4 score is 6 (IQR: 6.0, 9.0), indicating moderate depression/anxiety amongst participants. The majority of participants reported having depression/anxiety (*n* = 150, 75%), with 110 (64.5%) reporting moderate or severe depression/anxiety, while 50 (25%) reported no issues with mental health. Thirty‐three per cent (*n* = 66) of participants reported taking medication for anxiety and depression (citalopram and mirtazepine frequently prescribed), and 18.5% (*n* = 37) were prescribed antipsychotics (including quetiapine and olanzepine). Additionally, 26% (*n* = 52) were taking medications to treat substance dependence (including methadone and nicotine), and 10.5% (*n* = 21) were taking pain relief medications (such as codeine and co‐codamol).

### 3.5 Food Insecurity

**Figure 1:**
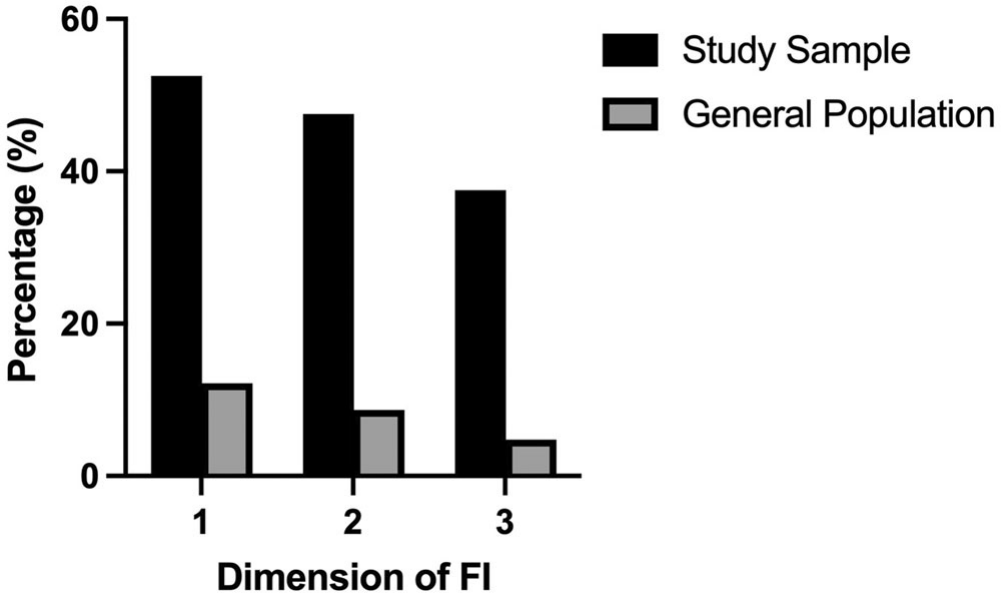
Comparison of food insecurity between participants and the UK general population using the screening questions used by the Food Foundation Food Insecurity Tracker (June 2024). 1: Ate smaller meals than usual or skipped meals altogether (%); 2: Felt hungry but did not eat (%); and 3: Did not eat for a whole day (%).

The median food security score was 4.5 (IQR: 0.0, 8.0), indicating low food security, with very low food security reported in nearly half of the participants (*n* = 88; 44.0%). In the past month, over half (*n* = 105, 52.5%) reported they ate smaller meals than usual or skipped meals altogether, nearly half of the participants (*n* = 95, 47.5%) reported feeling hungry but not eating, and over a third (*n* = 75, 37.5%) reported not eating for a whole day (Figure 1).

### 3.6 Dietary Intakes Compared With Recommendations

The median Diet Quality Score (DQS) was 8.0, (IQR: 6.0, 9.0), indicating overall low dietary quality. The median daily fruit intake was 0.4 portions (IQR: 0.2, 1.0) with 77.5% (*n* = 155) participants having less than one portion of fruit per day. The median daily vegetable intake was 0.5 portions per day (0.5, IQR: 0.2, 1.0), with 78% (*n* = 156) and 89.5% (*n* = 179) participants having less than one portion of vegetables or salad per day respectively. Additionally, intakes of wholegrain bread and cereal were low, with 80.5% (*n* = 161) and 78.5% (*n* = 157) having less than one portion per day, respectively. Generally, dietary intakes of fat, saturated fat, salt and sugar foods were similarly low, aside from sugar‐sweetened beverages, with 27% (*n* = 146) participants having at least one portion per day. Portions of protein were particularly low, with 99.5% (*n* = 199), 91% (*n* = 182) and 91.5% (*n* = 183) having less than one portion per day of white fish, poultry and red meat, respectively. Nearly half reported rarely or never drinking alcohol over the last week (47% [*n* = 94]), with 20.5% (*n* = 41) participants having less than 14 units, 7.5% (*n* = 15) having between 14 and 21 units, while a quarter (25% [*n* = 50]) consumed over 21 units over the last week.

Public Health England [13] shows the dietary intake of PEH living in temporary accommodation, using the 24‐h recall, compared with government dietary recommendations for energy, macronutrients, vitamins, and minerals [13]. Participant calorie intake was lower than the government‐recommended energy requirements for both men (1797 kcal/day; 71.9%) and women (1522.9 kcal/day; 76.1%), with free sugars contributing a higher proportion of calorie intake than recommended (59.3 g/day; 179.7%) for men and women (65.5 g/day; 242.6%), and lower fibre intakes for men (12.0 g/day; 41.0%) and women (13.5 g/day; 45.0%). Intakes of most vitamins and minerals were lower than recommended, notably vitamin D, iron, folate, calcium, magnesium and selenium (Table 2). However, intakes of chloride, thiamine, vitamin B12 and B6 exceeded recommendations (Table 2). Additionally, intakes of vitamin C were below the recommendations for men (38.4 mg/day; 95.9%) while they exceeded recommendations for women (114.7 mg/day; 286.9%). Of the 38 participants (19%) who reported drinking alcohol within the past 24 h, intakes nearly/exceeded the weekly recommended intake of 14 units (men; 106.9/day; 95.4%, and women; 197.9 g/day; 176.7%) [24].

**Table 2:** Dietary recommendations compared with national recommendations and general population intakes.

	Government dietary recommendations^a^		Participant median intake (IQR)		% RNI of median	
Nutrient	Female	Male	Female	Male	Female	Male
Macronutrients						
Energy (kcal)	2000	2500	1522.9 (1139.0, 2234.5)	1797.0 (1149.3, 2565.0)	76.1 (57.0, 111.7)	71.9 (46.0, 102.0)
Protein (g/day)	45.0	55.5	47.5 (25.7, 63.4)	64.7 (36.5, 98.1)	104.3 (56.4, 139.3)	116.5 (65.8, 175.7)
Fat (g/day)	78	97	47.2 (30.8, 77.7)	55.0 (27.5, 92.2)	60.5 (39.4, 99.6)	56.6 (28.4, 95.1)
Fat—SFA (g/day)	24	31	18.7 (11.5, 27.8)	18.5 (10.2, 32.2)	77.7 (49.8, 115.8)	59.7 (32.8, 103.8)
Fat—PUFA (g/day)	14	18	13. (5.9, 16.5)	14.3 (6.3, 22.1)	94.1 (42.2, 118.0)	79.2 (34.8, 122.5)
Fat—PUFA n3 (g/day)	—	—	1.3 (0.4, 1.9)	1.2 (0.5, 2.3)	—	—
Fat—PUFA n6 (g/day)	—	—	4.9 (2.8, 9.5)	6.7 (3.0, 12.4)	—	—
Fat—MUFA (g/day)	29	36	18.0 (10.7, 31.0)	18.9 (9.2, 32.9)	62.2 (36.9, 106.8)	52.5 (25.6, 91.2)
Carbohydrate (g/day)	267	333	191.5 (133.4, 266.6)	206.8 (132.8, 308.6)	71.7 (49.9, 99.9)	62.1 (40.0, 92.7)
Free Sugar (g/day)	27	33	65.5 (20.9, 109.6)	59.3 (24.6, 113.2)	242.6 (77.6, 406.0)	179.7 (74.4, 342.9)
Fibre (g/day)	30	30	13.5 (7.8, 18.2)	12.0 (7.3, 19.3)	45.0 (26.1, 60.7)	41.1 (24.8, 63.6)
Vitamins						
Vitamin A (mg/day)	600	700	584.3 (202.5, 962.3)	384.4 (133.2, 734.8)	97.4 (33.8, 160.4)	54.9 (19.0, 105.0)
Thiamin (mg/day)	0.8	1.0	1.3 (0.7, 1.9)	1.4 (0.7, 2.2)	156.3 (85.0, 238.8)	136.0 (70.0, 221.5)
Riboflavin (mg/day)	1.3	1.1	1.2 (0.7, 2.3)	1.4 (0.6, 2.4)	91.5 (50.0, 180.0)	110.0 (49.2, 186.5)
Niacin (mg/day)	16.5	13.2	13.9 (10.2, 25.5)	18.1 (9.5, 28.0)	180.2 (120.2, 286.4)	194.8 (103.1, 301.4)
Vitamin B6 (mg/day)	1.2	1.4	1.3 (0.8, 2.3)	1.4 (0.8, 2.6)	111.7 (67.5, 192.5)	102.9 (58.6, 188.6)
Vitamin B12 (mg/day)	1.5	1.5	3.5 (1.9, 6.8)	4.1 (2.2, 7.6)	232.7 (124.7, 455.3)	275.3 (146.3, 508.7)
Folate (mg/day)	200	200	176.1 (108.7, 334.6)	190.7 (103.3, 313.0)	88.1 (54.4, 167.3)	95.3 (51.6, 156.5)
Vitamin C (mg/day)	40	40	114.7 (41.2, 255.6)	38.4 (10.6, 107.8)	286.9 (103.1, 638.9)	95.9 (26.4, 269.4)
Vitamin D (mg/day)	10	10	1.8 (0.6, 8.1)	1.5 (0.5, 3.9)	18.4 (5.6, 81.0)	15.4 (4.7, 39.3)
Minerals						
Iron (mg/day)	14.8^a^	8.7	7.9 (5.2, 17.2)	8.1 (4.7, 16.4)	52.8 (34.6, 115.4)	93.3 (53.6, 188.9)
	8.7^b^		—	—	—	—
Calcium (mg/day)	700	700	567.3 (322.7, 1078.6)	706.2 (377.1, 1182.0)	81.0 (46.1, 154.1)	100.9 (53.9, 168.9)
Magnesium (mg/day)	270	300	186.8 (156.3, 329.5)	227.0 (140.1, 315.7)	69.2 (57.9, 122.0)	75.7 (46.7, 105.2)
Potassium (mg/day)	3500	3500	2271.5 (1667.0, 3178.6)	2192.2 (1385.7, 3123.0)	64.9 (47.6, 90.8)	62.6 (39.6, 89.2)
Zinc (mg/day)	7.0	9.5	5.0 (3.6, 9.3)	7.9 (4.4, 11.7)	71.4 (50.9, 132.3)	82.4 (45.8, 122.8)
Cooper (mg/day)	1.2	1.2	0.9 (0.6, 1.2)	0.9 (0.6, 1.5)	71.7 (51.7, 103.3)	78.3 (47.9, 124.6)
Iodine (mg/day)	140	140	99.7 (60.3, 240.1)	114.8 (48.3, 220.6)	71.2 (43.1, 171.5)	82.0 (34.5, 157.6)
Selenium (mg/day)	60	75	30.3 (15.4, 58.0)	38.9 (19.7, 62.0)	50.4 (25.7, 96.6)	51.8 (26.2, 82.7)
Phosphorus (mg/day)	550	550	897.8 (552.8, 1298.8)	975.2 (536.9, 1445.4)	163.2 (100.5, 236.1)	177.3 (97.6, 262.8)
Chloride (mg/day)	2500	2500	2220.9 (1300.1, 3273.6)	2426.5 (1396.5, 4138.3)	88.8 (52.0, 130.9)	97.1 (55.9, 165.6)
Sodium (g/day)	2.4	2.4	1.2 (0.7, 2.2)	1.7 (0.9, 2.6)	50.0 (30.3, 92.0)	70.9 (39.1 109.1)

Abbreviations: g/day, grams per day; IQR, interquartile range; kcal, calories; mg/day, milligrams per day; RNI, recommendation nutrition intake.

a Premenopausal.

b Postmenopausal.

A total of 42 participants were taking additional vitamin and/or mineral supplementation, including vitamin B complex, vitamins B1 (thiamine), B5 (pantothenic acid) and B12, vitamin C, iron and folate. Sensitivity analysis was conducted to remove these participants (Supporting Information S1: Table S1), revealing that the majority of percentage intakes were slightly lower and that riboflavin, vitamin B6 and calcium for men fell below the RNI (91.9%, 84.3% and 91.9%, respectively).

### 3.7 Predictors of Outcomes

Four multivariable regression models were conducted to assess predictors of diet quality, risk of malnutrition, food insecurity and HGS.

For diet quality being female was the only predictor of the DQS, with being female predicted lower DQS by −0.64 points (*p* = 0.035, 95% CIs: −1.23, −0.05) compared to being male (Supporting Information S1: Table S2).

For risk of malnutrition, assessed by MUST score, poorer mental health (PHQ‐4), high food insecurity score (USDA) and living with underweight predicted higher MUST score (Supporting Information S1: Tables S3 and S4). With each point increase, PHQ4 resulted in a 0.09 point increase in MUST score (95% CI: 0.04, 0.14 *p* < 0.001), similarly each point increase in USDA resulted in a 0.09 point increase in MUST score (95% CI: 0.03, 0.14 *p* = 0.002,). Living with underweight resulted in a 2.45 point increase in MUST score (95% CI: 1.79, 3.11 *p* < 0.001). Whereas living with a higher body weight predicted a lower MUST score. In participants living with overweight, there was a −0.80 point reduced in MUST (95% CI: −1.27, −0.33 *p* < 0.001), living with obesity class 1 a −1.14 point reduction (95% CI: −1.78, −0.49 *p* < 0.001) and living with obesity class 2 a −1.73 point reduction (95% CI: −2.87, −0.58 *p* < 0.001) in MUST score compared to those with a healthy weight.

When looking at functional strength, HGS was predicted by age, gender, BMI category and percentage muscle mass (Supporting Information S1: Tables S5 and S6). Older age and being female predicted lower HGS while having a higher BMI and living with a higher weight predicted higher HGS. For each year increase in age, there was a 0.21 point reduction in HGS (95% CI: −0.33, −0.10 *p* < 0.001) and being female resulted in a −7.84 point reduction in HGS (95% CI: −12.04, −3.64 *p* < 0.001). While for each percentage point increase in muscle mass resulted in a 0.26 increase in HGS (95% CI: 0.03, 0.50 *p* = 0.030) and for participants living with overweight, obesity class 1 and class 2 caused a 6.08, 5.99 and 10.36 point increase in HGS, respectively, compared to those with a healthy weight (95% CI: 2.66, 9.51; 95% CI: 0.83, 11.16; 95% CI: 2.13, 18.56, *p* = 0.005, respectively).

Finally, when looking at food insecurity, as assessed by USDA score, having poorer mental health predicted higher food insecurity, with every point increase in PHQ4 resulting in a 0.30 point increase in USDA score (*p* < 0.001, 95% CI: 0.19, 0.42). Participants living in catered accommodation resulted in reduced food insecurity, with catered accommodation causing a 2.01 point reduction in USDA score (*p* < 0.001, 95% CI: −2.97, −1.04) (Supporting Information S1: Tables S7 and S8).

## 4 Discussion

This is the first study to comprehensively assess the malnutrition risk of PEH living in temporary accommodation. Uniquely, this study used multiple methods of dietary assessment and anthropometry, including HGS, BIA scales, measurement of DQS, and nutrient composition, as well as measuring food security, to assess nutritional status. These results provide a novel, deeper understanding of nutrient intakes for PEH in temporary accommodation in London.

Our data demonstrates that PEH living in temporary accommodation were at high risk of malnutrition according to MUST [18]. The risk appeared to be related to step 3, disease prevalence [6] and/or prolonged periods of inadequate dietary intake [25], with low MUST scores being predicted by living with overweight and obesity, which are not detected as risks by MUST. This presents a concern with those living with higher body weight and identifying sarcopenic obesity [26, 27]. Furthermore, poor mental health and high food insecurity both predict high MUST scores and are prevalent in PEH, placing PEH at a potentially higher risk of malnutrition. Malnutrition risk could also be linked to low participant energy intakes compared with the general population [28]. With evidence showing that PEH reports frequent infection, poor skin, dental health [29], and poor access to healthcare services [6] and food provision [12], places PEH at significant risk of complications of undernutrition. Furthermore, despite the mean age of participants being 45.7 years, HGS readings were comparable with people aged 89, falling in lower centiles compared with the general population, indicating a risk of poorer health outcomes [30, 31]. The addition of HGS to screen for malnutrition risk resulted in an additional 7% of participants being identified. Our data showed that both age and female sex predicted lower HGS, while living with overweight or obesity and a higher percentage of muscle mass predicted higher HGS, which agrees with previous research [32]. Given these population‐specific complexities [33], a more sensitive nutritional screening tool, including HGS, would be of benefit in detecting nutritional risk amongst PEH [34].

Simultaneously, our data shows the prevalence of overweight and obesity, which is also reflected in other countries for PEH [35]; however, this was less than the general UK population [36].

Although the median visceral fat mass score was within the healthy range, 16% had visceral fat ≥ 12, indicating excess and possible risk of metabolic complications, which is reflected in other PEH [37]. Overweight and obesity for PEH can be linked to the hunger‐obesity paradox [38], the chronic state of hunger and obesity described amongst socially deprived groups [39]. Obesity is highly correlated with low income [40] and poor health outcomes [41]. This could infer that nutritional screening tools for PEH should incorporate the detection of obesity risk as well as malnutrition, as recommended for mental health populations [42].

Compared with the general population, participants reported experiencing higher levels of low or very low food insecurity (14.8% and 61.5%, respectively) [43]. Participants reported eating smaller meals or skipping meals altogether, feeling hungry but not eating, and not eating for a whole day over the previous 30 days. These were 4.3, 5.5 and 7.8 times higher than reported in the general population, respectively [43]. Importantly, living in catered accommodation predicted lower food insecurity, suggesting that temporary accommodation living in uncatered accommodations places PEH at greater risk of both lack of access to food and a higher risk of malnutrition. In addition, higher food insecurity appeared to impact negatively on mental health in a group already experiencing poor mental health. During study visits, a substantial proportion of participants anecdotally reported a lack of access to food, irrespective of the accommodation catering arrangements, which echoes other studies [44] and is concerning since 44% of hostels in the study offered food provision. Participants anecdotally reported frequently begging for food instead of accepting food donations related to not attending food banks as these have been reported to be long distances away on foot [12, 45]. Food insecurity screening also evoked strong emotional reactions amongst participants, possibly indicating participants' feelings of lack of autonomy and dignity surrounding dietary choices [46]. Based on the high prevalence of food insecurity amongst PEH, introducing routine screening in homeless care settings could be of merit to prevent undernutrition.

Participants had a low DQS compared with government guidance, which recommends higher intakes of fruits, vegetables and oily fish, and lower intakes of non‐milk extrinsic sugars and fats than the participants' [47]. Being female predicted lower dietary quality, which is consistent with previous research showing that women have poorer diet quality both in the general population [48] and those experiencing homelessness [49]. However, this is the first time in the homeless population to show quality was predicted by gender; this may be indicative of the small number of females within the sample, though warrants future research to explore this further. Our dietary recall data show that PEH were generally not meeting government dietary recommendations, and specifically had lower intakes of energy, protein, total fat, poly‐ and monounsaturated fat, carbohydrate and fibre. Data from the SFFFQ and recall data showed that dietary protein was sourced mainly from processed foods which are understood to be of poor quality [12]. This mirrors findings from studies of the diets of PEH [10, 14]. Our micronutrient data are comparable with a previous study examining dietary intakes in PEH, with similarly low intakes of vitamin A, iron, selenium and calcium [25], emphasising the disparity with the general population [50]. Our data also showed lower levels of vitamin D, similarly reflected within the general population [50]. Contrarily to previous data [25, 51], participants exceeded recommendations for thiamine, vitamin B12 and B6, and vitamin C in females; however, following sensitivity analysis of nutritional supplementation, men did not meet RNI for riboflavin, vitamin B6, and calcium from hostel food provision, indicating the role for continued prescription recommended as part of rehabilitation protocol for substance misuse [52]. Persistently low micronutrient intakes could clinically justify nutritional supplementation for PEH with or without substance dependence alongside food provision in temporary accommodation.

Over a third of participants consumed alcohol in excess of the government's maximal recommendations of 14 units per week, and this figure likely underestimates the extent of consumption. Concerningly, high alcohol intake amongst PEH is linked to higher rates of hospital admission and multimorbidity [53] and is linked to malnutrition [54]. Notably, a few (*n* = 3) participants presented with decompensated liver disease and ascites, masking malnutrition at the time of the study. Intervention is required to prevent malnutrition related to alcohol consumption.

Nearly two thirds of participants reported either moderately severe or severe mental illness, which is consistent with previous studies of PEH [55] with alcohol and drug misuse being reported the most common mental health disorders [53]. There was a high prevalence of depression and anxiety, and antidepressant treatment amongst participants. Additionally, a high proportion of participants were also prescribed antipsychotics, analgesia, treatment or substance dependence and sleeping medications, indicating poor mental health amongst participants [56]. However, a proportion of participants reported the absence of mental illness and variable compliance with medications, suggested to be influenced by substance misuse [55, 57]. Notably, the majority of the participants were male and, as such, may not have wished to appear to be vulnerable or asking for help, resulting in possible under‐reporting of symptoms [58]. Anecdotally some participants reported interactions between poor mental health and poor diet, including low self‐esteem and belief that they were undeserving of food compared to others, with previous data suggesting habitual disordered eating can act as a coping mechanism against hunger [44, 46]. These concerning remarks suggest that there may be a role for enhanced working with clinical specialist teams to support improved dietary outcomes related to mental health in PEH in temporary accommodation.

There are both strengths and limitations to this research that should be acknowledged. This is the first study to have comprehensively assessed malnutrition risk in PEH in temporary accommodation alongside data on dietary intake, food insecurity and mental health status.

Given that the study is cross‐sectional, long‐term studies are required to examine the nutritional status of PEH living in temporary accommodation over time. With the majority of participants living in temporary accommodation, being male, and white ethnicity, these results cannot be generalised to the wider homeless populations including rough‐sleepers, however, our data is comparable to demographic data of the PEH population as a whole in London [53, 59]. There were methodological limitations in the study related to the challenges linked to data collection amongst PEH. A single‐pass 24‐h recall was used for dietary intake analysis based on advice from hostel managers related to the transient nature of the hostel residents and the potential burden of the study visit. Furthermore, the analysis of dietary intakes could have been confounded by the varied and poorly understood catering provisions and charitable partnerships in each temporary accommodation. BIA measurements were not standardised, namely participants were not fasted nor screened for ascites or oedema, as visits were organised around when the residents were in the hostel. Additionally, the cooking facilities of the hostels were not formally assessed within the study, though they may have impacted food provision in addition to whether the hostels were catered or not. Despite these challenges, through close working with hostel managers to understand the practicalities of working with PEH, researchers were able to overcome many of the challenges; however, these should be considered when interpreting the data.

## 5 Recommendations

Reducing malnutrition risk and improving dietary intake for PEH living in temporary accommodation, although challenging, may be achievable. The following recommendations should be considered to help achieve this vision.

– To enable staff working in temporary accommodation to promote the dietary health of PEH, the development of policy, standards and guidelines regarding nutrition is strongly recommended to include optimised food environments, charity partnerships, and cooking facilities, as well as improved nutrition screening practices.
– Introduction of routine nutritional screening and monitoring could prompt more efficient referrals to and closer working with healthcare professionals, including dietitians, alongside specialist mental health, addiction and other clinical teams.
– Population‐specific nutritional screening tools could be developed to account for the unique dietary considerations of PEH, including sensitive markers, such as handgrip strength, obesity, food insecurity and mental health, which can help identify those at greater nutritional risk.
– Temporary accommodations could strengthen food aid charity partnerships and be supported to prioritise improved dietary quality of food and catering services.
– For PEH living in temporary accommodation with identified nutritional risk, prompt referral to dietetics services is recommended.
– In the absence of consistent food provision and dietary intake for PEH, prescription of oral nutritional supplements and vitamin and mineral supplementation could be considered by dietitians and other prescribers to improve nutritional status.
– Standards surrounding cooking facilities, storage, and catering in temporary accommodations should be developed with stakeholders to enhance access to food of a consistently high dietary quality.
– Further analysis of dietary habits and qualitative research examining dietary health beliefs and behaviours of staff and residents could support the coproduction of tailored and realistic dietary interventions for PEH living in temporary accommodation.

## 6 Conclusion

PEH residing in temporary residences experience dietary health inequalities, which are currently poorly detected. This population group are at greater risk of malnutrition, poor dietary quality and food insecurity than the general population and therefore interventions to address these are urgently needed. Compounded by severe mental illness and substance misuse, PEH residing in temporary accommodation are at greater risk of poor health outcomes related to their diet. Improved nutritional screening practices, including increased frequency and detection of obesity, substance misuse, mental illness and food security, could serve to expedite referrals to clinical teams and prevent malnutrition onset. Although multifactorial and complex in presentation, malnutrition amongst PEH appears preventable. Improved partnerships with charitable services could address the high levels of food insecurity. Findings from this study could help to inform policy‐makers about the dietary needs of PEH and support them in setting standards for temporary accommodation, which could enhance their services to provide tailored dietary intervention for this at‐risk population group.

## Author Contributions

A.B. and H.S. conceived the study and contributed to the study and survey design and methodology. A.B. was responsible for the oversight of the study. H.S. led the recruitment of participants and conducting of the study. A.B. and V.V. were responsible for the data analysis. All authors contributed to data interpretation, and the writing of the manuscript. All authors contributed to critical revision of the manuscript and gave final approval.

## Conflicts of Interest

The authors declares no conflicts of interest.

### 1 Peer Review

The peer review history for this article is available at https://www.webofscience.com/api/gateway/wos/peer-review/10.1111/jhn.70024.

## Transparency Declaration

The authors confirm that this manuscript is an accurate, honest, and transparent account of the study being reported. The reporting of this research is compliant with STROBE^2^ guidelines. The authors confirm that no important aspects of the study have been omitted and that any discrepancies from the study as planned by UCL and FEAST with US have been explained.

## Supporting information

Supporting information.

## Data Availability

Deidentified participant data that underlie the results reported in this article will be made available on publication and ending 5 years after publication. Proposals should be made to the corresponding author and will require a data access agreement.
