# Supplementary material for: Nutrition Status of People Experiencing Homelessness Residing in Temporary Accommodation in London
Source: J Hum Nutr Diet. 2025 Feb 9;38(1):e70024. doi: 10.1111/jhn.70024 (PMC11808289; doi:10.1111/jhn.70024)
Supplement: Supplementary file 1 — Supporting information. [file JHN-38-0-s001.docx]

**Table S1: Recommended nutritional intakes to national recommendations with and without supplementations**

| **Nutrient** | **% RNI of Median with supplementation** | | **% RNI of Median without supplementation** | |
| --- | --- | --- | --- | --- |
|  | **Female** | **Male** | **Female** | **Male** |
| **Vitamins** |  |  |  |  |
| Vitamin A (mg/day) | 97.4 (33.8, 160.4) | 54.9 (19.0, 105.0) | 99.7 (30.8, 140.4) | 44.3 (14.0, 90.5) |
| Thiamin (mg/day) | 156.3 (85.0, 238.8) | 136.0 (70.0, 221.5) | 152.5 (67.2, 220.6) | 105.0 (56.5, 189.0) |
| Riboflavin (mg/day) | 91.5 (50.0, 180.0) | 110.0 (49.2, 186.5) | 71.9 (42.9, 147.1) | 91.9 (42.1, 152.1) |
| Niacin (mg/day) | 180.2 (120.2, 286.4) | 194.8 (103.1, 301.4) | 172.9 (119.1, 282.9) | 180.0 (91.5, 281.7) |
| Vitamin B6 (mg/day) | 111.7 (67.5, 192.5) | 102.9 (58.6, 188.6) | 106.3 (56.3, 190.8) | 84.3 (46.1, 173.0) |
| Vitamin B12 (mg/day) | 232.7 (124.7, 455.3) | 275.3 (146.3, 508.7) | 206.3 (97.3, 455.8) | 237.7 (104.7, 467.8) |
| Folate (mg/day) | 88.1 (54.4, 167.3) | 95.3 (51.6, 156.5) | 76.9 (46.3, 138.2) | 79.0 (39.9, 118.7) |
| Vitamin C (mg/day) | 286.9 (103.1, 638.9) | 95.9 (26.4, 269.4) | 175.9 (55.1, 650.3) | 72.6 (14.4, 188.3) |
| Vitamin D (mg/day) | 18.4 (5.6, 81.0) | 15.4 (4.7, 39.3) | 17.2 (4.4, 52.6) | 10.6 (3.0, 31.9) |
| **Minerals** |  |  |  |  |
| Iron (mg/day) | 52.8 (34.6, 115.4) | 93.3 (53.6, 188.9) | 46.4 (26.6, 64.1) | 85.3 (43.7, 168.2) |
|  | - | - | - | - |
| Calcium (mg/day) | 81.0 (46.1, 154.1) | 100.9 (53.9, 168.9) | 78.6 (45.8, 162.6) | 91.9 (47.5, 171.4) |
| Magnesium (mg/day) | 69.2 (57.9, 122.0) | 75.7 (46.7, 105.2) | 68.3 (54.1, 119.6) | 71.0 (41.6, 104.0) |
| Potassium (mg/day) | 64.9 (47.6, 90.8) | 62.6 (39.6, 89.2) | 59.7 (44.0, 100.6) | 59.7 (33.2, 87.9) |
| Zinc (mg/day) | 71.4 (50.9, 132.3) | 82.4 (45.8, 122.8) | 58.7 (43.9, 123.9) | 72.5 (37.9, 112.9) |
| Cooper (mg/day) | 71.7 (51.7, 103.3) | 78.3 (47.9, 124.6) | 68.3 (39.8, 83.8) | 71.7 (36.5, 122.3) |
| Iodine (mg/day) | 71.2 (43.1, 171.5) | 82.0 (34.5, 157.6) | 65.6 (39.6, 154.7) | 67.2 (22.7, 143.7) |
| Selenium (mg/day) | 50.4 (25.7, 96.6) | 51.8 (26.2, 82.7) | 41.2 (23.7, 91.7) | 47.7 (23.0, 73.9) |
| Phosphorus (mg/day) | 163.2 (100.5, 236.1) | 177.3 (97.6, 262.8) | 150.0 (92.4, 238.0) | 163.2 (88.1, 262.3) |
| Chloride (mg/day) | 88.8 (52.0, 130.9) | 97.1 (55.9, 165.6) | 84.3 (43.8, 133.8) | 97.1 (49.6, 167.7) |
| Sodium (g/day) | 50.0 (30.3, 92.0) | 70.9 (39.1 109.1) | 48.1 (27.9, 92.7) | 71.6 (35.6, 110.9) |

| **Table S2: Univariable regression output, exploring the relationships with Diet Quality Score (DQS)** | | | |
| --- | --- | --- | --- |
|  | Coefficient | 95% CI | p-value |
| DQS |  |  |  |
| Age | 0.00 | [-0.02, 0.02] | 0.877 |
| Gender |  |  |  |
| Male | (ref) |  |  |
| Female | -0.64 | [-1.23,-0.05] | 0.035 |
| Ethnicity (grouped) |  |  |  |
| Other | 0.27 | [-0.17, 0.72] | 0.224 |
| White | (ref) |  |  |
| BMI Categories |  |  | 0.813 |
| Underweight | 0.06 | [-0.70, 0.81] |  |
| Health Weight | (ref) |  |  |
| Overweight | -0.14 | [-0.67, 0.38] |  |
| Obesity Class 1 | 0.36 | [-0.38, 1.09] |  |
| Obesity Class 2 | 0.07 | [-1.22, 1.36] |  |
| PHQ4 score | 0.03 | [-0.02, 0.09] | 0.220 |
| USDA score | 0.04 | [-0.01, 0.10] | 0.136 |
| Hostel catering |  |  |  |
| Not catered | (ref) |  |  |
| Catered | 0.11 | [-0.33, 0.54] | 0.629 |
| Fibre AOAC | -0.01 | [-0.04, 0.01] | 0.209 |

To identify the optimal multivariable model to predict the consumption of DQS, a stepwise regression model was run. Gender was the only predictor variables that remained in the model. The model results can be viewed in the table above.

| **Table S3: Univariable regression output, exploring the relationships with MUST score** | | | |
| --- | --- | --- | --- |
|  | Coefficient | 95% CI | p-value |
| MUST score |  |  |  |
| Age | 0.01 | [-0.01, 0.03] | 0.405 |
| Gender |  |  |  |
| Male | (ref) |  |  |
| Female | -0.22 | [-0.90, 0.46] | 0.527 |
| Ethnicity (grouped) |  |  |  |
| Other | -0.27 | [-0.78, 0.24] | 0.293 |
| White | (ref) |  |  |
| BMI Categories |  |  | <0.001 |
| Underweight | 2.38 | [1.66, 3.10] |  |
| Health Weight | (ref) |  |  |
| Overweight | -0.90 | [-1.41,-0.40] |  |
| Obesity Class 1 | -1.17 | [-1.87,-0.46] |  |
| Obesity Class 2 | -1.45 | [-2.69,-0.22] |  |
| HGS (best score) | -0.02 | [-0.04, 0.01] | 0.138 |
| DQS | 0.05 | [-0.11, 0.21] | 0.555 |
| PHQ4 score | 0.11 | [0.05, 0.17] | <0.001 |
| USDA score | 0.14 | [0.07, 0.20] | <0.001 |
| Hostel catering |  |  |  |
| Not catered | (ref) |  |  |
| Catered | -0.42 | [-0.91, 0.08] | 0.098 |
| Energy (kcal) | -0.00 | [-0.00, 0.00] | 0.461 |
| Protein | -0.01 | [-0.01,-0.00] | 0.012 |

To identify the optimal multivariable model to predict the consumption of MUST score, a stepwise regression model was run. Age, USDA score, PHQ4 score, and BMI Categories remained predictor variables in the model. The final multivariable regression model is shown below.

| **Table S4: Multivariable regression output, exploring the relationships with MUST Score** | | | |
| --- | --- | --- | --- |
|  | Coefficient | 95% CI | p-value |
| MUST score |  |  |  |
| Age | 0.02 | [0.01, 0.04] | 0.005 |
| USDA score | 0.09 | [0.03, 0.14] | 0.002 |
| PHQ4 score | 0.09 | [0.04, 0.14] | <0.001 |
| BMI Categories |  |  | <0.001 |
| Underweight | 2.45 | [1.79, 3.11] |  |
| Healthy Weight | (ref) |  |  |
| Overweight | -0.80 | [-1.27,-0.33] |  |
| Obesity Class 1 | -1.14 | [-1.78,-0.49] |  |
| Obesity Class 2 | -1.73 | [-2.87,-0.58] |  |

| **Table S5: Univariable regression output, exploring the relationships with USDA Score** | | | |
| --- | --- | --- | --- |
|  | Coefficient | 95% CI | p-value |
| USDA score |  |  |  |
| Age | -0.03 | [-0.08, 0.01] | 0.165 |
| Gender |  |  |  |
| Male | (ref) |  |  |
| Female | -0.81 | [-2.27, 0.64] | 0.273 |
| Ethnicity (grouped) |  |  |  |
| Other | 0.36 | [-0.72, 1.44] | 0.511 |
| White | (ref) |  |  |
| BMI Categories |  |  | 0.257 |
| Underweight | -0.26 | [-2.07, 1.56] |  |
| Health Weight | (ref) |  |  |
| Overweight | -1.43 | [-2.71,-0.16] |  |
| Obesity Class 1 | -0.35 | [-2.12, 1.43] |  |
| Obesity Class 2 | -1.39 | [-4.51, 1.72] |  |
| HGS (best score) | 0.05 | [-0.00, 0.10] | 0.054 |
| DQS | 0.26 | [-0.08, 0.60] | 0.136 |
| PHQ4 score | 0.34 | [0.22, 0.46] | <0.001 |
| Hostel catering |  |  |  |
| Not catered | (ref) |  |  |
| Catered | -2.44 | [-3.44,-1.43] | <0.001 |
| Energy (kcal) | -0.00 | [-0.00, 0.00] | 0.159 |

To identify the optimal multivariable model to predict consumption of USDA score, a stepwise regression model was run. HGS (best), hostel catering (catered vs not), and BMI categories were the predictor variables that remained in the model. The final multivariable regression model is shown below.

| **Table S6: Multivariable regression output, exploring the relationships with USDA Score** | | | |
| --- | --- | --- | --- |
|  | Coefficient | 95% CI | p-value |
| USDA score |  |  |  |
| HGS (best score) | 0.05 | [-0.00, 0.09] | 0.057 |
| PHQ4 score | 0.30 | [0.19, 0.42] | <0.001 |
| Hostel catering |  |  |  |
| Not catered | (ref) |  |  |
| Catered | -2.01 | [-2.97,-1.04] | <0.001 |
| BMI Categories |  |  |  |
| Underweight | -0.19 | [-1.80, 1.41] | 0.092 |
| Health Weight | (ref) |  |  |
| Overweight | -1.50 | [-2.64,-0.37] |  |
| Obesity Class 1 | 0.02 | [-1.58, 1.62] |  |
| Obesity Class 2 | -1.63 | [-4.43, 1.17] |  |
|  |  |  |  |

| **Table S7: Univariable regression output, exploring the relationships with HGS Score** | | | |
| --- | --- | --- | --- |
|  | Coefficient | 95% CI | p-value |
| HGS (best score) |  |  |  |
| Age | -0.20 | [-0.32,-0.08] | 0.001 |
| Gender |  |  |  |
| Male | (ref) |  |  |
| Female | -10.70 | [-14.28,-7.12] | <0.001 |
| Ethnicity (grouped) |  |  |  |
| Other | -1.02 | [-3.90, 1.85] | 0.484 |
| White | (ref) |  |  |
| BMI Categories |  |  | 0.211 |
| Underweight | -0.58 | [-5.40, 4.24] |  |
| Health Weight | (ref) |  |  |
| Overweight | 3.21 | [-0.18, 6.60] |  |
| Obesity Class 1 | 3.02 | [-1.70, 7.75] |  |
| Obesity Class 2 | 5.37 | [-2.91,13.65] |  |
| PHQ4 score | 0.17 | [-0.17, 0.51] | 0.324 |
| MUST score | -0.60 | [-1.39, 0.19] | 0.138 |
| USDA score | 0.36 | [-0.01, 0.73] | 0.054 |
| Muscle mass percent | 0.23 | [0.07, 0.39] | 0.005 |
| Energy (kcal) | 0.00 | [-0.00, 0.00] | 0.515 |
| Protein | 0.05 | [0.01, 0.08] | 0.006 |

A stepwise regression model was run to identify the optimal multivariable model to predict consumption of HGS score. Age, gender, BMI categories, protein, muscle mass (percent), USDA score, and energy (kcal) remained predictor variables in the model. The final multivariable regression model is shown below.

| **Table S8: Multivariable regression output, exploring the relationships with HGS Score** | | | |
| --- | --- | --- | --- |
|  | Coefficient | 95% CI | p-value |
| HGS (best score) |  |  |  |
| Age | -0.21 | [-0.33,-0.10] | <0.001 |
| Gender |  |  |  |
| Male | 0.00 |  |  |
| Female | -7.84 | [-12.04,-3.64] | <0.001 |
| Ethnicity |  |  |  |
| Non-white | -2.38 | [-5.00, 0.23] | 0.074 |
| White | (ref) |  |  |
| BMI Categories |  |  | 0.005 |
| Underweight | -2.64 | [-6.97, 1.69] |  |
| Health Weight | (ref) |  |  |
| Overweight | 6.08 | [2.66, 9.51] |  |
| Obesity Class 1 | 5.99 | [0.83,11.16] |  |
| Obesity Class 2 | 10.36 | [2.13,18.59] |  |
| Protein | 0.05 | [0.00, 0.09] | 0.031 |
| Muscle mass percent | 0.26 | [0.03, 0.50] | 0.030 |
| USDA score | 0.30 | [-0.03, 0.63] | 0.073 |
| Energy (kcal) | -0.00 | [-0.00, 0.00] | 0.095 |
|  |  |  |  |
